# Staged Minimally Invasive Management of Prolapsed Submucosal Myoma With Severe Bleeding: A Case Report of Uterine Artery Embolization, Relugolix Therapy, and Transcervical Resection

**DOI:** 10.7759/cureus.105870

**Published:** 2026-03-26

**Authors:** Toma Fukuda, Hyo Kyozuka, Yumiko Kitago, Hajime Odajima, Yasuhisa Nomura

**Affiliations:** 1 Obstetrics and Gynaecology, Ohta Nishinouchi Hospital, Koriyama, JPN; 2 Pathology, Ohta Nishinouchi Hospital, Koriyama, JPN

**Keywords:** minimally invasive therapy, prolapsed myoma, relugolix, staged treatment, submucosal fibroid, transcervical resection, uterine artery embolization

## Abstract

We report the case of a 34-year-old woman with a prolapsed submucosal myoma who presented with severe genital bleeding. She was initially treated at a referring hospital, where she required a red blood cell transfusion for acute blood loss before transfer to our institution. After temporary stabilization, uterine artery embolization (UAE) was performed as an early minimally invasive intervention for the prolapsed myoma. Embolization was carried out using 500-700 μm tris-acryl gelatin microspheres (Embosphere; Merit Medical Systems Inc., South Jordan, USA). Oral relugolix (40 mg/day) was started several days after UAE and continued for approximately four months. Serial magnetic resonance imaging (MRI) demonstrated progressive tumor shrinkage from 41.6 × 23.6 mm before treatment to 24.8 × 10.8 mm and subsequently to 10.7 × 5.0 mm. After sufficient reduction in tumor size, transcervical resection (TCR) was performed successfully. The residual pedunculated lesion was resected with minimal blood loss in a seven-minute procedure. Histopathological examination showed hyalinized and degenerative leiomyoma with foreign body-type reaction, consistent with prior embolization-related change. The postoperative course was uneventful, and normal menstruation resumed after completion of medical therapy. This case suggests that a staged minimally invasive strategy combining UAE, relugolix therapy, and TCR may be an effective treatment option for prolapsed submucosal myoma with severe bleeding.

## Introduction

Uterine fibroids are common benign uterine tumors in women of reproductive age and may cause heavy menstrual bleeding, dysmenorrhea, pelvic pressure, and reproductive symptoms [1]. Among symptomatic cases, prolapsed submucosal fibroids can present with acute bleeding, pain, and technical difficulty in immediate transvaginal management, depending on tumor size, cervical dilatation, and patient condition [1]. Current management of symptomatic uterine fibroids emphasizes individualized and minimally invasive treatment whenever feasible [2]. Available options include medical therapy, uterine artery embolization (UAE), hysteroscopic resection, laparoscopic hysterectomy, and robot-assisted surgery, with treatment selection guided by symptom severity, anatomic findings, urgency, and patient-specific circumstances [3-5]. In selected cases, UAE can reduce fibroid perfusion and function as a bridge to subsequent minimally invasive surgery [2,5]. Relugolix, an oral gonadotropin-releasing hormone receptor antagonist, has also emerged as an important medical option for reducing fibroid-related bleeding and fibroid volume. A staged approach may be particularly useful when immediate definitive transvaginal or hysteroscopic treatment is not ideal in the acute setting, such as after recent hemodynamic compromise or when less invasive interval treatment may improve the feasibility of definitive resection. We report a case of prolapsed submucosal myoma with severe bleeding that was successfully managed using a staged minimally invasive strategy consisting of UAE, relugolix therapy, and subsequent transcervical resection (TCR).

## Case presentation

**Figure 1:**
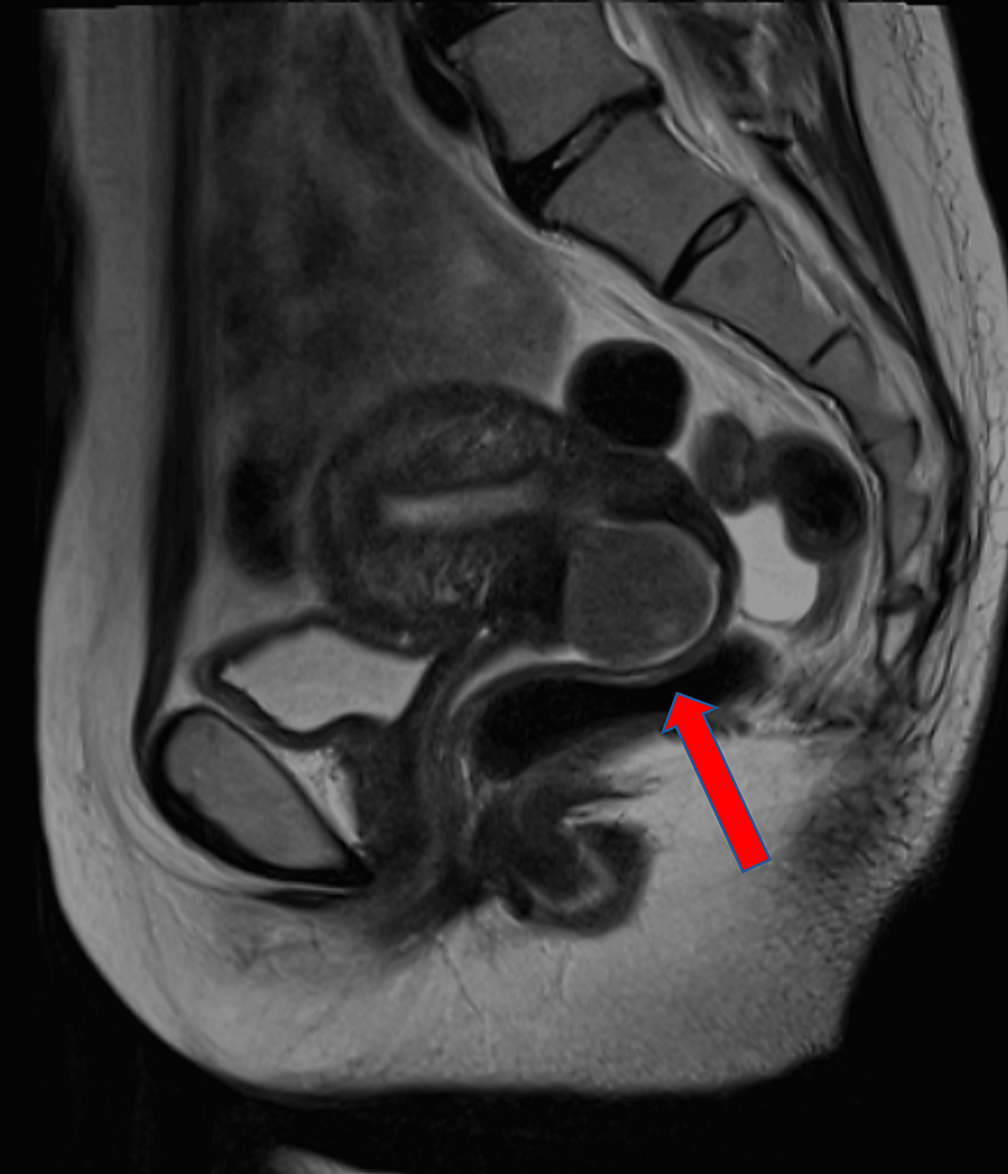
Sagittal pelvic MRI of a prolapsed submucosal myoma Sagittal pelvic MRI showing a pedunculated submucosal myoma prolapsing through the cervical canal. The red arrow indicates the protruding portion of the myoma beyond the external cervical os. MRI: magnetic resonance imaging

A 34-year-old woman was transferred to our hospital after being diagnosed with a prolapsed myoma at a referring hospital. She had developed massive genital bleeding with hypotension and received four units of red blood cells before transfer. At the time of transfer, active bleeding had improved after transfusion. Her blood pressure was 120/50 mmHg, heart rate was 80 beats/min, and laboratory testing showed hemoglobin of 8.1 g/dL (reference range: 11.6-14.8 g/dL), fibrinogen of 250 mg/dL (reference range: 150-400 mg/dL), prothrombin time-international normalized ratio (PT-INR) of 1.04 (reference range: 0.8-1.2), and activated partial thromboplastin time (APTT) of 26.7 seconds (reference range: 24-37 seconds). Gynecologic examination revealed a prolapsed mass consistent with a pedunculated submucosal myoma extending through the cervical canal, with associated genital bleeding. However, a complete detailed transvaginal assessment was limited at presentation because of recent heavy bleeding and the need to prioritize hemodynamic stabilization. In addition, immediate transvaginal manipulation was considered less suitable because the patient had no prior history of sexual intercourse. Magnetic resonance imaging (MRI) performed at the referring hospital demonstrated a pedunculated prolapsed submucosal myoma measuring 41.6 × 23.6 mm. The stalk measured 7.7 mm, and the lesion protruded approximately 7 mm beyond the external cervical os (Figure 1). The differential diagnosis included cervical myoma, prolapsed endometrial polyp, and, less likely, a malignant polypoid lesion; however, the imaging findings and subsequent histopathology were consistent with a prolapsed submucosal leiomyoma. Because uterine preservation was considered an important factor in treatment planning, and because bleeding had stabilized after transfusion but the patient remained at risk of recurrent hemorrhage, UAE was performed shortly after transfer as minimally invasive treatment for the prolapsed myoma. Immediate transvaginal or hysteroscopic resection was not considered ideal at presentation because the patient had recently experienced hemodynamic instability due to massive bleeding, and urgent definitive local intervention was considered less appropriate before stabilization. In addition, the prolapsed pedunculated lesion still had substantial volume at that stage, which was expected to make immediate minimally invasive resection more technically demanding.

**Figure 2:**
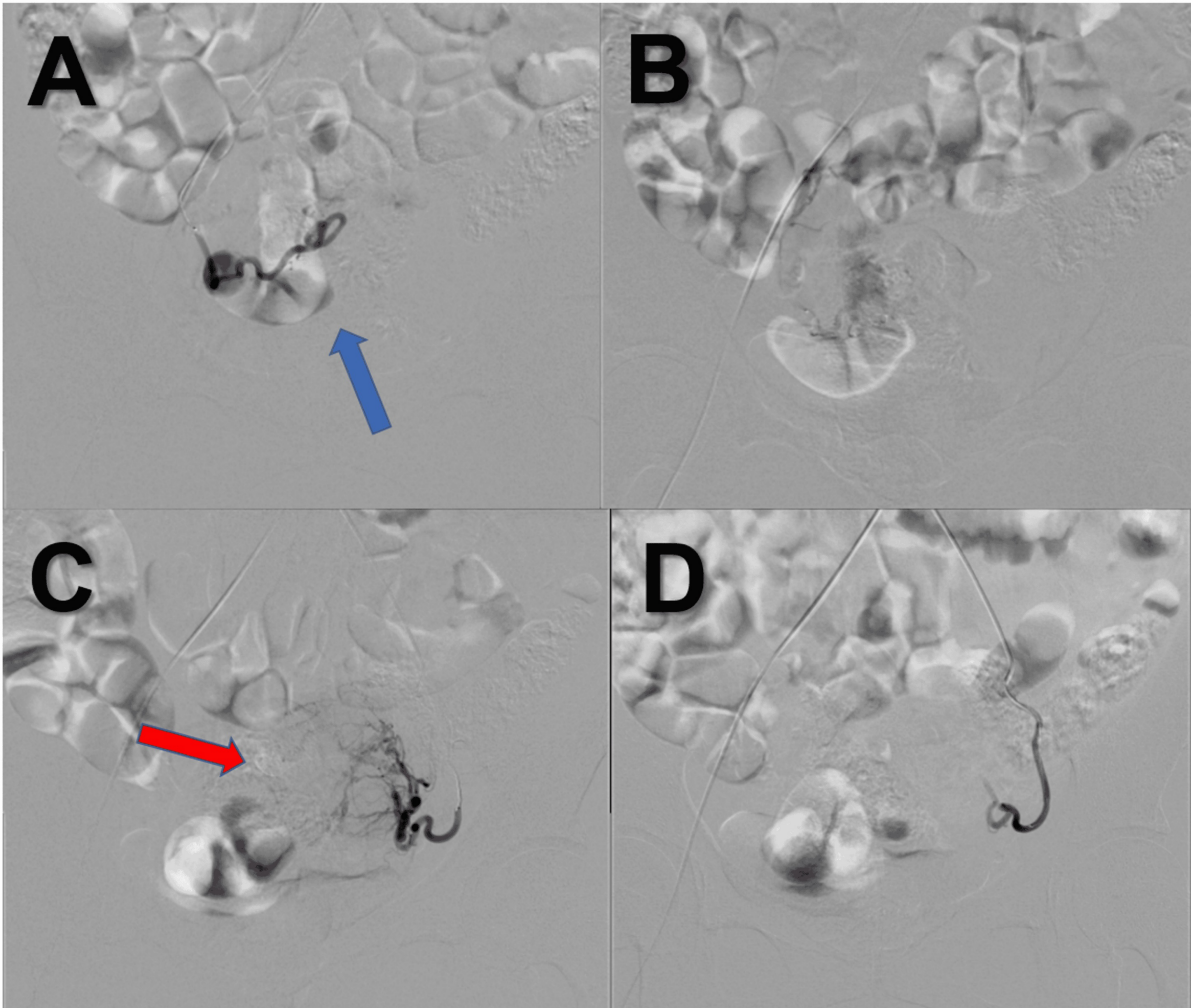
Angiographic findings before and after UAE (A) Right uterine artery angiography before embolization. The blue arrow indicates uterine perfusion supplied by the right uterine artery. (B) Right uterine artery angiography after embolization, showing the disappearance of the uterine perfusion seen in panel (A). (C) Left uterine artery angiography before embolization. The red arrow indicates uterine perfusion supplied by the left uterine artery. (D) Left uterine artery angiography after embolization, showing the disappearance of the uterine perfusion seen in panel (C). UAE: uterine artery embolization

As in our previously reported institutional UAE protocol [5], the procedure was performed via the right common femoral artery under local anesthesia. A 5-Fr sheath was inserted, and selective angiography was performed using a 5-Fr diagnostic catheter (MORI catheter; Medikit Co. Ltd., Tokyo, Japan). A 2.2/2.9-Fr microcatheter (Carnelian ER; Tokai Medical Products, Kasugai City, Japan) was then advanced for selective catheterization of the uterine arteries. A nonionic iodinated contrast medium (iopamidol 300 mg I/mL) was used, with a total contrast volume of 100 mL. Embolization was performed bilaterally using 500-700 μm tris-acryl gelatin microspheres (Embosphere; Merit Medical Systems Inc., South Jordan, USA), and the endpoint was marked reduction of uterine artery flow (Figure 2).

**Figure 3:**
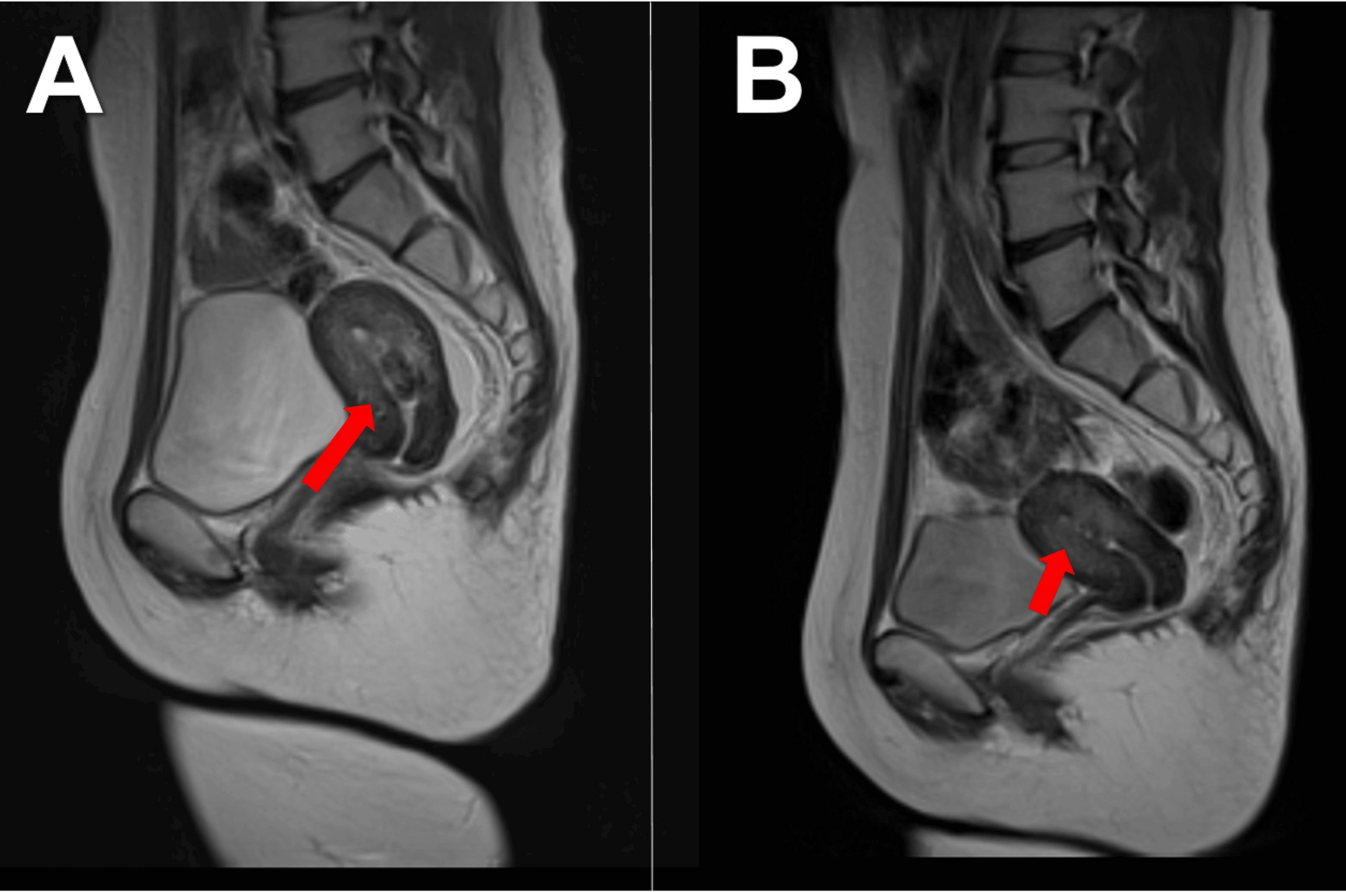
Serial MRI demonstrating progressive shrinkage of the prolapsed submucosal myoma after UAE and relugolix therapy (A) Sagittal pelvic MRI obtained approximately two months after UAE. The red arrow indicates the reduced submucosal myoma. (B) Sagittal pelvic MRI obtained approximately six months after UAE, immediately prior to TCR. The red arrow indicates further shrinkage of the submucosal myoma. UAE: uterine artery embolization; MRI: magnetic resonance imaging; TCR: transcervical resection

After UAE, hemoglobin remained stable at 8.3 g/dL, and no additional transfusion was required. Oral relugolix at a dose of 40 mg/day was initiated several days after UAE and continued for approximately four months. It was started after confirmation of hemostatic stabilization, with the aim of promoting further myoma shrinkage and facilitating less invasive definitive resection. No clinically significant adverse effects of relugolix were documented during treatment. Add-back hormonal therapy was not used, and bone density monitoring was not performed because short-term treatment was planned; additionally, these assessments are not routinely conducted at our institution in this setting. Follow-up MRI performed approximately two months after UAE showed tumor shrinkage to 24.8 × 10.8 mm, and later MRI demonstrated further reduction to 10.7 × 5.0 mm (Figure 3).

**Figure 4:**
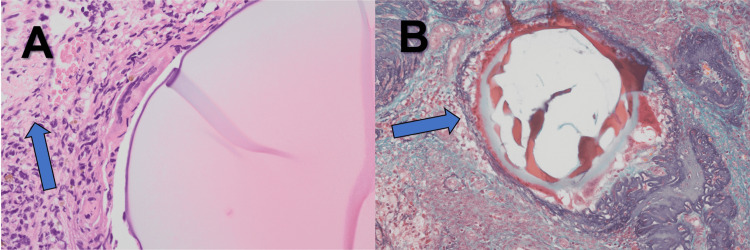
Histopathological findings of the resected myoma (A) H&E staining (×400) showing hyalinized and degenerative leiomyoma (blue arrow). (B) Elastica-Masson staining (×200) showing mildly dilated vessel-like structures containing eosinophilic material, with foreign body-type giant cell-like cells and histiocyte-like cells along the periphery (blue arrow), suggesting a reaction to the embolic material. H&E: Hematoxylin and eosin

After sufficient shrinkage had been confirmed, TCR was performed. A small residual pedunculated myoma was identified at the 4 o’clock direction between the uterine isthmus and lower uterine body. The lesion was resected in fragments using a loop electrode (Olympus, Tokyo, Japan). The operative time was seven minutes, and blood loss was minimal. Histopathological examination revealed a hyalinized and degenerative leiomyoma. Elastica-Masson (EM) staining showed mildly dilated vessel-like structures containing eosinophilic material, with foreign body-type giant cell-like cells and histiocyte-like cells along the periphery, suggesting a reaction to the embolic material (Figure 4).

The postoperative course was uneventful. Normal menstruation resumed approximately three months after completion of relugolix therapy. The patient had had regular menstrual cycles before treatment, and dysmenorrhea and abnormal uterine bleeding resolved after treatment. Overall, her menstrual symptoms normalized during follow-up.

The clinical course is summarized in Table 1.

**Table 1:** Clinical course and staged minimally invasive management of the prolapsed submucosal myoma MRI: magnetic resonance imaging; PT-INR: prothrombin time-international normalized ratio; APTT: activated partial thromboplastin time

Clinical stage	Findings/intervention
At the referring hospital	Massive genital bleeding with hypotension; four units of red blood cells were transfused.
Transfer to our hospital	Hemodynamically stabilized after transfusion; hemoglobin 8.1 g/dL, fibrinogen 250 mg/dL, PT-INR 1.04, and APTT 26.7 seconds.
Initial MRI	Prolapsed pedunculated submucosal myoma, 41.6 × 23.6 mm, with a 7.7-mm stalk and protrusion beyond the external cervical os.
Initial assessment and treatment planning	Immediate definitive transvaginal or hysteroscopic resection was considered less suitable because of recent hemodynamic instability, lesion bulk, limited transvaginal assessment at presentation, and the importance of uterine preservation in treatment planning.
Early intervention	Uterine artery embolization was performed via the right common femoral artery using 500-700 μm tris-acryl gelatin microspheres (Embosphere; Merit Medical Systems Inc., South Jordan, USA).
After uterine artery embolization	Hemoglobin remained stable at 8.3 g/dL, and no additional transfusion was required.
Medical therapy	Oral relugolix 40 mg/day was started several days after uterine artery embolization and continued for approximately four months.
Follow-up MRI after uterine artery embolization	MRI performed approximately two months later showed tumor shrinkage to 24.8 × 10.8 mm.
Preoperative follow-up MRI	Later MRI demonstrated further shrinkage to 10.7 × 5.0 mm.
Definitive treatment	Transcervical resection was performed after sufficient shrinkage was confirmed; operative time was seven minutes and blood loss was minimal.
Pathology	Histopathological examination showed hyalinized and degenerative leiomyoma with findings suggestive of a reaction to the embolic material.
Outcome	Uneventful postoperative recovery; normal menstruation resumed approximately three months after completion of relugolix therapy.

## Discussion

This case highlights the usefulness of a staged minimally invasive strategy for a prolapsed submucosal myoma associated with severe bleeding. After initial stabilization with transfusion at the referring hospital, UAE was selected to reduce vascular supply to the lesion and lower the risk of recurrent hemorrhage. Subsequent relugolix therapy was associated with progressive tumor shrinkage, which allowed definitive treatment by TCR with minimal blood loss and a short operative time.

Prolapsed submucosal fibroids are often managed by transvaginal or hysteroscopic removal; however, immediate definitive intervention may be difficult in patients with recent hemodynamic compromise or when local manipulation is not readily feasible [1,6]. In the present case, UAE was performed after bleeding had temporarily improved, not as a rescue procedure during ongoing shock, but as an early minimally invasive therapeutic step to facilitate safer subsequent management. This distinction is important in understanding the role of UAE in this treatment sequence.

Serial MRI demonstrated continued reduction in fibroid size after UAE and during relugolix therapy, suggesting a combined effect of devascularization and hormonal suppression. A prior report has described a similar staged concept in which UAE combined with relugolix was followed by hysteroscopic surgery for fibroid management [2]. In our case, the lesion decreased from a prolapsed pedunculated myoma to a small residual lesion that could be removed hysteroscopically with minimal operative burden.

Relugolix, an oral gonadotropin-releasing hormone receptor antagonist, has become an important medical option for reducing fibroid-related bleeding and fibroid volume [1,2,7]. In this patient, relugolix likely contributed to sustained shrinkage after the initial reduction in blood flow induced by UAE, thereby improving the feasibility of a minimally invasive definitive procedure [2,7]. The progressive decrease in lesion size on serial imaging supports the clinical utility of combining medical therapy with a staged interventional approach in selected patients.

The final TCR was completed safely with minimal bleeding and in only seven minutes, suggesting that this staged strategy reduced procedural complexity and operative burden. Histopathological findings of hyalinization, degeneration, and foreign body-type reaction were also consistent with prior embolization-related change.

At our institution, treatment options for symptomatic uterine fibroids have expanded to include UAE, hysteroscopic surgery, total laparoscopic hysterectomy, and robot-assisted hysterectomy, allowing management to be tailored according to tumor morphology, symptom severity, urgency, and procedural considerations [3,4]. This broader therapeutic spectrum reflects an effort to increase minimally invasive treatment choices for patients with uterine fibroids. The present case further extends that approach by showing that even a prolapsed submucosal myoma with severe bleeding can be managed successfully using UAE, medical shrinkage, and TCR without resorting to more invasive surgery.

In addition, our prior institutional experience has shown that UAE can be incorporated into staged treatment strategies for complex fibroid cases, including situations in which the reduction of bleeding risk is clinically important before definitive surgery [5]. The present case differs in that the definitive procedure was TCR rather than laparoscopic surgery, but it similarly demonstrates how UAE can broaden therapeutic flexibility in challenging presentations.

This report is limited by its single-case design. Nevertheless, it suggests that a combination of UAE, relugolix therapy, and subsequent TCR may be a practical, minimally invasive option for selected patients with prolapsed submucosal myoma and severe bleeding, particularly when immediate definitive transvaginal intervention is not ideal.

## Conclusions

A staged minimally invasive strategy using UAE, relugolix therapy, and TCR may be a practical treatment option for prolapsed submucosal myoma with severe bleeding. In this case, the approach allowed hemostatic control, tumor shrinkage, and definitive treatment without the need for more invasive surgery. This treatment sequence may be particularly useful in selected patients in whom immediate definitive transvaginal or hysteroscopic management is not ideal. Although further studies are needed to confirm its broader applicability, this case suggests that combining interventional, medical, and hysteroscopic treatment can expand minimally invasive options for challenging fibroid presentations.
